# Epidemic of Scurvy in Edinburgh

**Published:** 1847-06

**Authors:** 


*Epidemic of Scurvy in Edinburgh.*—A very singular epidemic of scurvy has broken out among the railway labourers in the neighborhood of Edinburgh. On the day we write this, no less than 92 cases of the disease, in all its different stages, may be seen in the Royal Infirmary, special wards having been opened for their reception. In the autumn of last year a similar epidemic occurred among the prisoners of the General Penitentiary at Perth. Both these epidemics have fortunately found an able historian in Professor Christison, who, at the last meeting of the Medico-Chirurgical Society, read an elaborate memoir, abounding in novel views of the etiology and mode of treatment of the disease. We shall have the pleasure of presenting our readers with this valuable contribution to medical science in our next number.—*Ibid.*

